# The Fees of Practitioner and Consultant

**Published:** 1920-04-17

**Authors:** 


### TIED HOUSES IN SURGICAL PRACTICE.

### The Fees of Practitioner and Consultant.

A short while ago a young and ambitious surgeon, impoverished through service abroad during the war, returned to the provincial town in which he intended to practice surgery. After his long absence work did not come to him so rapidly as to relieve his financial anxieties. In these circumstances he was approached by a strong firm of practitioners in the town, who stated that they were about to take in an additional partner, and further that it was their intention to ask a highly qualified and competent surgeon to join them, unless our friend were willing to make a verbal and private agreement with them to return to the firm one-third of all the surgical fees which he should receive from patients belonging to their practice; while they, on their part, would undertake to collect the fees and to carry out the after-treatment of any patients who might have undergone operations. With this proposition he found it necessary to agree as a matter of self-preservation. But he informs us that he has felt a little uncomfortable ever since lest the compact might be regarded as in some way discreditable or lacking in unrightness and honour.

### No New Thing.

Now the division of fees between practitioner and consultant—dichotomy, as it is called—is no new thing; it has become already, we believe, an established and recognised custom in some parts of the world, and evidence is not wanting that it is taking root in our own soil. For these reasons it is worth while to consider the respective advantages and drawbacks which may be inherent in business methods of this kind as attached to medical practice. We know that a considerable proportion of the price paid by a private individual for a motor-car, for example, represents a commission to the retailer, the payment being countenanced by the manufacturer of the article. But there is this difference, that the public is fully aware of the commission, whereas in the refund of a part of his fee by a consultant to the practitioner who sends the patient to him, the public is unaware of the transaction. And, as concerns ethics, this appears to be the crux of the matter.

### The Practice of Dichotomy.

The practice of dichotomy also carries with it certain other disadvantages; the undesirability of a practitioner being tied to one particular consultant is one of them. For no man can be absolutely competent in every branch of surgery. He may be excellent in the fields of abdominal and gynæcological work, and yet relatively deficient in skill when required to deal with, let us say, any orthopædic case, or with one needing an operation for the cure of a deflected nasal septum or a chronic frontal sinusitis. Yet the lure of the refunded fee may lead the practitioner to employ his usual surgeon for such purposes in the place of a man who is better qualified to deal with them.

Such a consideration is sure to prove more than a hypothetical disadvantage, in spite of the fact that efficiency pays, and that the medical practitioner who always does the best that is possible for his patient will, in the long run, meet with greater satisfaction and prosperity than fall to the lot of his rival who allows more immediate and mercenary influences to control his conduct.

### True Working Agreements.

On the other hand, working agreements between consultants and practitioners, based upon what foundations they may be, are undoubtedly of value in several respects. Not only, by ensuring to the would-be consultant a certain measure of practice and a tangible prospect of the nucleus of an income, do they encourage early specialism, but they enable the surgeon to know his practitioner and the practitioner to know his surgeon, while they greatly facilitate co-operation and that form of team-work which the war has shown to be of the utmost value in dealing with the problems of disease. Moreover, there is a manifest advantage in an arrangement which will prompt a doctor to refer those of his patients who require surgical treatment to a specialist surgeon rather than to the tender mercies of a "surgical partner" whose time is largely taken up with the calls of general practice, whose technical experience is strictly limited, and whose capacity, in consequence of these drawbacks, is bound to be restricted.

Indeed, it is arguable that the mutual benefits to be derived from a close and continued inter-participation of knowledge, and from a free and unembarrassed interchange of thought between the surgeon and the general practitioner will outweigh the disadvantages which are attached to an arrangement based upon the principles of dichotomy. For the evils of dichotomy are not essential and ineradicable. So long as discrimination is used, and the first rule of efficiency in treatment, so as to secure the best for the patient, is not lost to sight, and so long as the compact is made openly, good rather than evil may accrue.

### A Fundamental Distinction.

When, on the other hand, the demand is made by a doctor to a consultant for a refund of a percentage of his fee, without observance of these modifying and refining considerations, without any view to a lasting co-operation and mutual desire to be helpful, it merely amounts to a kind of blackmail, and is not to be tolerated for an instant either by the consultants or by the public. The distinction, perhaps, is, on a superficial and passing glance, not very plainly discernible, and yet it is, we feel, a real and fundamental one. And if dichotomy is to become a common custom in this country, as present indications seem to suggest that it will be, it is to be hoped that a careful scrutiny will be observed so that it may be confined

**Tied Houses in Surgical Practice**—(*continued*).

within the limits of its legitimate boundaries, and above all that, it shall emerge from the shadows and no longer be transacted in secrecy and in fear of public opinion.

**[We have received this stimulating paper from an old and valued correspondent; and the force of his reasoning is undeniable. At the same time, we do not share his belief that "dichotomy" is to be encouraged or even tolerated in this country, though we admit that it has certain advantages (to the patient) as set forth in his argument. In the case of our correspondent and of practitioners equally imbued with the lofty ideals of professional honour which we know that he cherishes, little, if any, harm would accrue—less perhaps than the countervailing benefits; but there are numbers of men and women in the medical profession of much less integrity. To admit the principle would not only allow these latter a cloak for mercenary and disreputable traffic, but would distinctly put temptation in the way of many others who do at present practice reasonably ethical conduct. Not only so, it would deal a severe blow to the faith of the general public in their medical attendants; for our correspondent's plea that "dichotomy" should be openly admitted and practised is a counsel of perfection which is probably unattainable.—Editor, THE HOSPITAL.]**

